# What do HTA agencies need for generating health-related quality of life evidence? Findings from a global survey

**DOI:** 10.1017/S0266462326103602

**Published:** 2026-02-27

**Authors:** Annushiah Vasan Thakumar, Paula Lorgelly, Louise Longworth, Lucila Rey-Ares, Fredrick Purba, Dominik Golicki, Federico Augustovski, Kim Rand, Rosalie Viney, Nick Bansback, Nan Luo

**Affiliations:** 1 School of Pharmacy, Faculty of Health and Medical Sciences, Taylor’s University, Malaysia; 2 School of Population Health and Department of Economics, University of Auckland, New Zealand; 3 Arrow Health Economics, UK; 4 Patient and Health Impact, Pfizer Argentina, Argentina; 5 Department of Clinical and Health Psychology, Faculty of Psychology, Universitas Padjadjaran, Indonesia; 6 Department of Experimental and Clinical Pharmacology, Medical University of Warsaw, Poland; 7 Health Technology Assessment and Health Economics Department, Institute for Clinical Effectiveness (IECS-CONICETUBA), Argentina; 8 Health Services Research Unit, Akershus University Hospital, Lørenskog, Norway; 9 Maths in Health, Klimmen, The Netherlands; 10 Centre for Health Economics Research and Evaluation, University of Technology Sydney, Australia; 11 School of Population and Public Health, The University of British Columbia, Canada; 12 Saw Swee Hock School of Public Health, National University of Singapore, Singapore

**Keywords:** global survey, health technology assessment, health-related quality of life, health-state utility, HT A policy, MAUI, preference elicitation

## Abstract

**Objectives:**

The overall aim is to understand the practices, views, and needs of health technology assessment (HTA) practitioners worldwide regarding the use of health-related quality of life (HRQoL) data for generating cost-effectiveness evidence.

**Methods:**

We invited HTA practitioners in sixty countries to complete an online survey on their perspectives on the measurement and valuation of health. We performed descriptive analyses of the overall sample, examined response differences across six regions, and pooled responses to open-ended questions for content analysis.

**Results:**

A total of 238 individuals from 45 countries completed the survey, with a mean response number per country of 5.28 (SD: 4.45). Most responses came from public sector employees (seventy-two percent), and ninety percent were involved in cost-effectiveness-related work. The top three most frequently used utility instruments were EQ-5D, SF-6D, and EQ-5D-Y, and the elicitation methods were time trade-off, visual analogue scale, and standard gamble. Health-state preferences of the general public from another country were more frequently used than the preferences of the local public. Common data quality issues were poor sample representativeness and a small sample size of utility data. In Asia and Western Europe, the top-voted research priority was developing utility instruments that capture both healthcare and social care impact. In four regions, developing utility instruments for children was the second-highest research priority.

**Conclusions:**

The survey addressed important knowledge gaps regarding current practices in measuring and valuing HRQoL in HTA and provided insights into HTA practitioners’ views on instruments, methods, and data-related challenges and needs for generating HRQoL evidence.

## Introduction

Health technology assessments (HTA) provide a comprehensive framework for integrating evidence of economic, social, and health consequences into decision-making (1). Globally, healthcare systems increasingly rely on HTA to guide resource allocation (2–4). In HTA, cost-effectiveness analysis (CEA) of new health technologies using quality-adjusted life years (QALYs) as the health effects measure is the most recommended form of economic evaluation (2;5). However, estimating QALYs is technically challenging as it requires measuring and valuing patients’ health-related quality of life (HRQoL). Consequently, analysts often rely on HRQoL and health-state utility (HSU, valuation of HRQoL) data from the literature, which can be limited in both quantity and quality.

HSU data can be estimated directly by describing a specific health condition using vignettes and capturing preferences through elicitation methods such as the time trade-off (TTO) and discrete choice experiments (DCE). However, the indirect method of using standardized preference-weighted HRQoL instruments, or multi-attribute utility instruments (MAUIs) such as EQ-5D to obtain HSU data is more commonly used (6). In recent years, there has been significant progress in developing new methods and instruments for measuring and valuing HRQoL. For example, there is an increasing application of DCE in the valuation of HRQoL (7). Since DCE can be used in self-administered online surveys, it significantly lowers costs and shortens data collection timelines compared to traditional preference elicitation methods, which entail interviewer administration. Consequently, it has enabled more MAUIs to be developed, including disease-specific instruments such as the QLU-C10D and FACT-8D (8–10). Other examples include “bolt-on” research to enhance existing EQ-5D instruments (11;12) and the EQ-HWB instrument to broaden the ‘Q’ in QALY to cover both health and well-being (13). A bolt-on dimension refers to a supplementary item, or set of items, capturing specific aspects of health-related quality of life that may not be adequately represented by the original five dimensions of the EQ-5D instrument (14). Despite their attractive features, these new methods and instruments also have disadvantages. For instance, DCE and widely used traditional preference elicitation methods, such as TTO, have been shown to have poor agreement (15;16). Additionally, the primary challenge of DCEs arises during the analysis, as it requires careful assessment of whether respondents have completed the tasks appropriately. The use and testing of newer instruments may not be extensive, resulting in a lack of psychometric evidence and utility weights to inform decision-making. Given the wide range of methods and instruments available, and the proliferation of new ones, it remains unclear how existing instruments and methods are being used, and whether or when the new ones will be adopted by HTA practitioners for routine use.

HTA agencies or bodies (hereafter referred to as ‘HTA agencies’ for simplicity) are authoritative entities or divisions responsible for HTA evidence assessment and/or appraisal of health technologies. Although their size, capacity, and mandate vary, HTA agencies play a pivotal role in using HTA evidence to inform healthcare decisions. Moreover, because of their authority, HTA agencies’ practices and views on evidence generation methods significantly influence practice and therefore, are highly valuable to researchers. One source of information for understanding HTA agencies’ views and preferences is the methods guide they publish (17). However, this approach is suboptimal, particularly if the interest concerns HRQoL measurement and valuation methods. First, published methods guides may become outdated as practices and views continuously evolve, yet these guides are not updated frequently. Second, the guides for certain methodological aspects may be ambiguous or missing. Third, some recommendations in methods guides may not reflect unanimous opinions within HTA agencies. Last but not least, the absence of published methods guides by many HTA agencies limits their use as a resource for understanding these agencies’ practices.

An alternative way to understand HTA agencies’ views and preferences regarding HRQoL measurement and valuation is to survey HTA agency personnel responsible for preparing or reviewing HTA dossiers. This approach offers the advantage of obtaining first-hand, contemporary, and detailed information, which could be very useful for HRQoL researchers to set their priorities. However, to the best of our knowledge, such an approach has not been explored before. Our primary objective was to understand HTA practitioners’ views on and needs for HRQoL-related methods and instruments. Our secondary objective was to understand their current practices and views on the availability and quality of HRQoL data and research priorities.

## Methods

We conducted a cross-sectional online survey of HTA agency personnel from April 2023 to January 2024. In order to achieve broad geographic coverage of this hard-to-reach population, we used purposive sampling and invited survey respondents through the professional networks of the diverse international study team. The study received ethics approval from the Institutional Review Board of National University of Singapore (IRB number: NUS-IRB-2022–426).

### Sampling and recruitment design

We employed a two-stage recruitment procedure. In the first stage, we identified target HTA agencies, defined as independent organizations or governmental divisions authorized to generate and/or review HTA evidence for market access or reimbursement decisions at the health system level. We targeted surveying HTA agencies in fifty countries. We used the search strategy adopted by Kennedy-Martin et al., who identified forty-six countries with existing HTA agencies in 2019 (18). We complemented this strategy with other sources, including the Gear4Health database (19), ISPOR’s Pharmacoeconomic Guidelines Around the World database (17), the WHO Health Technology Assessment and Health Benefit Package Survey 2020/2021 webpage (4), the INAHTA Members List (20), and consulted with EuroQol Group members and colleagues for additional input.

In the second stage, we identified members of the EuroQol Group or their acquaintances as recruiters for the countries we intended to survey. Recruiters sent invitations with country-specific survey links to potential respondents working in the target HTA agencies. Alternatively, they identified a contact person within each HTA agency to extend the survey invitations internally. A survey administrator monitored survey yields and prompted recruiters and/or contact persons to send reminders to potential respondents weekly for at least three consecutive weeks after the survey commenced in each country.

### Participants

Rather than surveying official representatives of the target agencies, we sought to recruit all personnel involved in handling CUA or HRQoL evidence, specifically, individuals responsible for reviewing, generating, and/or using QALY-based evidence. Additionally, those involved in HTA-related work but not directly handling QALY-related tasks were also invited to participate if interested.

The inclusion criteria for the survey were: 1) being an employee of an HTA agency (e.g., governmental or public agency, division, body, or committee) whose agency responsibilities included evaluating or appraising health technologies for the purpose of listing/delisting, reimbursement, or pricing/repricing at the national level, or being a contracted professional, consultant, or advisor to such HTA organization(s); 2) being able to understand the English survey form and complete open-ended questions in their language of choice; and 3) providing informed consent.

### Survey form

After obtaining consent, participants were invited to complete an electronic survey form powered by Qualtrics anonymously at their convenience. The survey form was developed by the study team, aiming to create a short survey that can be self-completed by most respondents in no more than 20 minutes. An iterative question drafting procedure was used, with multiple rounds of pilot-testing conducted with personnel from HTA agencies in Singapore, Indonesia, Canada, England, Norway, Australia, New Zealand, Colombia, and Argentina until the development goal was achieved.

The final survey started off with screening questions followed by a consent-taking question. Eligible and consenting participants were invited to complete six sections of questions revolving around their experience with and opinions on Utility Instruments, Elicitation Methods, Data Source, Data Quality and Appropriateness, and Research Topics of Importance. Further information on the survey form can be found in the Supplementary Material.

We used a four-point Likert-type response scale to assess frequency in Sections One to Four (“never”/ “not sure,” “occasionally,” “often,” and “very often”). In Sections One to Five, text fields were provided for respondents to explain their responses and elaborate on other methods, instruments, concerns, or research topics not covered in the survey.

### Statistical analysis

Descriptive analyses were performed for responses to closed-ended survey questions. For Likert-type questions in Sections One to Four, we first used the mode (or median if no or multiple modes were present) as the summary of the responses for each country, and then used the median of relevant country summaries as the summary of the responses for six regions (Commonwealth – Australia/Canada/New Zealand/United Kingdom, Western Europe, Central/Eastern Europe, Asia, Latin America, and Middle East/Africa).

To analyze the nominated research priorities in Section Five, we calculated a country-specific importance score for each research topic by averaging the scores from all respondents in the relevant country. Once the importance scores for all countries were available, a regional score was calculated by averaging scores from relevant countries in the region and a global score was calculated by averaging the regional scores. More information on how respondent-level scores were assigned is provided in the Supplementary Material.

All statistical analysis was performed using STATA v14 (StataCorp, College Station, TX, USA). All qualitative responses to the open-ended questions were collated and systematically reviewed. Non-English responses were translated using a forward-backward approach. We then undertook a structured content analysis, involving iterative coding and categorization of the data, to identify recurrent themes, underlying concepts, and salient patterns across respondents. This analytic approach facilitated a deeper understanding of the key issues raised by participants and allowed us to synthesize their viewpoints into coherent thematic domains (21).

## Results

### Sample characteristics

Of the sixty countries enlisted and approached, the survey was distributed in forty-nine countries. The remaining eleven countries were excluded for various reasons, including non-responsiveness (*N* = 2), infancy of HTA (*N* = 2), or the non-use of CUA (*N* = 3), declination/technical difficulty (*N* = 2), or political turmoil (*N* = 2). In forty-five of these countries, we received at least one completed survey (median: 4; interquartile range: 2 to 6), while in the remaining four countries, there were zero responses despite multiple follow-ups. Supplementary Table 1 outlines the distribution of responses and reasons for non-responses from the eleven countries approached. In total, 238 individuals in 45 countries and 65 HTA agencies completed and submitted the survey (Table 1 and Supplementary Table 1). Overall, the majority of the responses came from public sector employees (71.9 percent) and had at least four years of experience in HTA work (58.8 percent). Additionally, 81.1 percent of the respondents reported the presence of QALY estimation guidance in their work setting, and 89.5 percent were involved in QALY-related work responsibilities. More than half (61.3 percent) reviewed QALY-based cost-effectiveness evidence submitted by industry or contractors, and 91.2 percent performed HTA work at the national level. Of the 238 respondents surveyed, 25 did not have work responsibilities related to reviewing, generating, and/or using QALY-based cost-effectiveness evidence. These respondents mainly came from Vietnam (*N* = 10), Slovenia (*N* = 3), Austria (*N* = 2), Colombia (*N* = 2), and South Africa (*N* = 2).

**Table 1.** Characteristics of respondents (*N* = 238)

	Region, *n*(%)						Total, *n*(%)
	Common-wealth	Western Europe	Central/ Eastern Europe	Asia	Latin America	Middle East/ Africa	
Public sector employee							
Yes	21 (61.8)	24 (77.4)	19 (76.0)	77 (81.1)	20 (52.6)	10 (66.7)	171 (71.9)
No	13 (35.2)	7 (22.6)	6 (24.0)	18 (19.0)	18 (47.4)	5 (33.3)	67 (28.2)
Contracted professional							
Yes	17 (50.0)	14 (45.2)	8 (32.0)	33 (34.7)	26 (68.4)	5 (33.3)	103 (43.3)
No	17 (50.0)	17 (54.8)	17 (68.0)	62 (65.3)	12 (31.6)	10 (66.7)	135 (56.7)
Experience with HTA (years)							
Less than a year	3 (8.8)	3 (9.7)	6 (24.0)	31 (32.6)	1 (2.6)	5 (33.3)	49 (20.6)
1–3 years	6 (17.7)	3 (9.7)	7 (28.0)	19 (20.0)	12 (31.6)	2 (13.3)	49 (20.6)
4–6 years	7 (20.6)	3 (9.7)	2 (8.0)	9 (9.5)	9 (23.7)	0 (0)	30 (12.6)
7–9 years	0 (0)	0 (0)	1 (4.0)	7 (7.4)	1 (2.6)	1 (6.7)	10 (4.2)
10 years or more	18 (52.9)	22 (71.0)	9 (36.0)	29 (30.5)	15 (39.5)	7 (46.7)	100 (42.0)
Gender							
Female	17 (51.5)	23 (74.2)	17 (70.8)	55 (57.9)	17 (44.7)	7 (46.7)	136 (57.6)
Male	16 (48.5)	8 (25.8)	7 (29.2)	40 (42.1)	21 (55.3)	8 (53.3)	100 (42.4)
Age group (years)							
≤ 25	1 (2.94)	0 (0)	0 (0)	0 (0)	0 (0)	0 (0)	1 (0.4)
26–35	10 (29.4)	3 (9.7)	9 (36.0)	34 (35.8)	8 (21.1)	2 (13.3)	66 (27.7)
36–45	9 (26.5)	8 (25.8)	6 (24.0)	39 (41.1)	11 (29.0)	4 (26.7)	77 (32.4)
46–55	9 (26.5)	14 (45.2)	6 (24.0)	17 (17.9)	13 (34.2)	8 (53.3)	67 (28.2)
56–65	5 (14.7)	6 (19.4)	2 (8.0)	3 (3.2)	5 (13.2)	1 (6.7)	22 (9.2)
≥ 66	0 (0)	0 (0)	2 (8.0)	2 (2.1)	1 (2.6)	0 (0)	5 (2.1)
Education attainment							
Bachelors	2 (5.9)	0 (0)	0 (0)	11 (11.6)	0 (0)	1 (6.7)	14 (5.9)
Masters	15 (44.1)	7 (22.6)	12 (48.0)	38 (40.0)	24 (63.2)	4 (26.7)	100 (42.0)
Doctorate	17 (50.0)	24 (77.4)	13 (52.0)	46 (48.4)	14 (36.8)	9 (60.0)	123 (51.7)
Decline to disclose	0 (0)	0 (0)	0 (0)	0 (0)	0 (0)	1 (6.7)	1 (0.4)
Professional identity							
Health economist	31 (91.2)	21 (67.7)	9 (36.0)	29 (30.5)	19 (50.0)	9 (60.0)	118 (50.0)
Pharmacist	0 (0)	1 (3.2)	9 (36.0)	26 (27.4)	3 (7.9)	1 (6.7)	40 (16.8)
Public health workforce	0 (0)	4 (12.9)	3 (12.0)	21 (22.1)	6 (15.8)	0 (0)	34 (14.3)
Clinician/Medical doctor	0 (0)	1 (3.2)	3 (12.0)	2 (2.1)	1 (2.6)	2 (13.3)	9 (3.8)
Epidemiologist	0 (0)	0 (0)	0 (0)	3 (3.16)	6 (15.8)	0 (0)	9 (3.8)
Statistician	0 (0)	1 (3.2)	0 (0)	4 (4.21)	0 (0)	0 (0)	5 (2.1)
Other	3 (8.8)	3 (9.7)	1 (4.0)	10 (10.5)	3 (7.8)	3 (20.0)	23 (9.7)
Presence of QALY estimation guidance							
Yes	31 (91.2)	28 (90.3)	17 (68.0)	85 (89.5)	21 (55.3)	11 (73.3)	193 (81.1)
No	3 (8.8)	3 (9.7)	8 (32.0)	10 (10.5)	17 (44.7)	4 (26.7)	45 (18.9)
QALY-based responsibilities							
Yes	33 (97.1)	27 (87.1)	22 (88.0)	84 (88.4)	34 (89.5)	13 (86.7)	213 (89.5)
No	1 (2.9)	4 (12.9)	3 (12.0)	11 (11.6)	4 (10.5)	2 (13.3)	25 (10.5)
Role							
Review Industry	31 (91.2)	17 (54.8)	18 (72.0)	52 (54.7)	17 (44.7)	11 (73.3)	146 (61.3)
Review Public	13 (38.2)	17 (54.8)	9 (36.0)	47 (49.5)	22 (57.9)	8 (53.3)	116 (48.7)
Primary study	17 (50.0)	10 (32.3)	2 (8.0)	34 (35.8)	8 (21.1)	6 (40.0)	77 (32.4)
Recommend method	9 (26.5)	7 (22.6)	2 (8.0)	12 (12.6)	5 (13.2)	5 (33.3)	40 (16.8)
None of the above	1 (2.9)	4 (12.9)	2 (8.0)	7 (7.4)	8 (21.1)	1 (6.7)	23 (9.7)
Level of HTA work							
National	33 (97.1)	30 (96.8)	25 (100)	89 (93.7)	30 (79.0)	10 (66.7)	217 (91.2)
Regional provincial or state-level	3 (8.8)	9 (29.0)	1 (4.0)	15 (15.9)	3 (7.9)	6 (40.0)	37 (15.6)
Hospital	1 (2.9)	3 (9.7)	3 (12.0)	7 (7.4)	7 (18.4)	0 (0)	21 (8.8)
Health plan	1 (2.9)	5 (16.1)	2 (8.0)	13 (13.7)	6 (15.8)	5 (33.3)	32 (13.5)
Health technology appraised							
Pharmaceuticals	34 (100)	23 (74.2)	23 (92.0)	72 (75.8)	35 (92.1)	12 (80.0)	199 (83.6)
Medical devices	17 (50.0)	18 (58.1)	8 (32.0)	39 (41.1)	23 (60.5)	9 (60.0)	114 (47.9)
Vaccines	10 (29.4)	15 (48.4)	6 (24.0)	29 (30.5)	20 (52.6)	6 (40.0)	86 (36.1)
Diagnostics	14 (41.2)	17 (54.8)	4 (16.0)	25 (26.3)	22 (57.9)	6 (40.0)	88 (37.0)
Surgical procedures	11 (32.4)	19 (61.3)	2 (8.0)	18 (19.0)	10 (26.3)	4 (26.7)	64 (26.9)
Public health professionals	3 (8.8)	11 (35.5)	3 (12.0)	23 (24.2)	5 (13.2)	2 (13.3)	47 (19.8)
Other	2 (5.9)	6 (19.4)	3 (12.0)	9 (9.5)	2 (5.3)	0 (0)	22 (9.2)
Therapeutic area							
Oncology	26 (76.5)	14 (45.2)	19 (76.0)	67 (70.5)	29 (76.3)	12 (80.0)	167 (70.2)
Cardiovascular Disease	16 (47.1)	15 (48.4)	19 (76.0)	40 (42.1)	16 (42.1)	5 (33.3)	111 (46.6)
Diabetes/ Hypertension/Dyslipidaemia	9 (26.5)	12 (38.7)	11 (44.0)	43 (45.3)	13 (34.2)	7 (46.7)	95 (39.9)
Respiratory Disease	9 (26.5)	0 (0)	3 (12.0)	21 (22.1)	6 (15.8)	3 (20.0)	42 (17.7)
Musculoskeletal/ Rheumatology	8 (23.5)	8 (25.8)	6 (24.0)	7 (7.4)	11 (29.0)	0 (0)	40 (16.8)
Gynaecology/Obstetrics	1 (2.9)	3 (9.7)	1 (4.0)	6 (6.3)	3 (7.9)	3 (20.0)	17 (7.1)
Infections Disease/ HIV/ AIDS	1 (2.9)	6 (19.4)	0 (0)	17 (17.9)	10 (26.3)	6 (40.0)	40 (16.8)
Neurology	6 (17.7)	3 (9.7)	2 (8.0)	7 (7.4)	7 (18.4)	5 (33.3)	30 (12.6)
Psychiatric Disorders/ Substance Abuse	2 (5.9)	6 (19.4)	3 (12.0)	7 (7.4)	1 (2.6)	1 (6.7)	20 (8.4)
Gastrointestinal Disease	7 (20.6)	4 (12.9)	0 (0)	7 (7.4)	4 (10.5)	0 (0)	22 (9.2)
Endocrine	4 (11.8)	2 (6.5)	3 (12.0)	7 (7.4)	3 (7.9)	0 (0)	19 (8.0)
Surgery/Transplantation	2 (5.9)	4 (12.9)	1 (4.0)	6 (6.3)	1 (2.6)	0 (0)	14 (5.9)
Urology/Nephrology	0 (0)	4 (12.9)	2 (8.0)	7 (7.4)	2 (5.3)	0 (0)	15 (6.3)
Dermatology	5 (14.7)	3 (9.7)	0 (0)	2 (2.1)	1 (2.6)	3 (20.0)	14 (5.9)

*Note*: *Government employee:* In terms of employment type, the respondent is an employee of a governmental or public agency, division, body, or committee whose responsibilities include evaluation or appraisal of health technologies for the purpose of listing/delisting, reimbursement, or pricing/repricing at the national level; *Contracted professional:* The respondent is a contracted professional, consultant, or advisor of the above-mentioned HTA organisation(s); *QALY-based Responsibilities:* The respondents’ responsibilities in the above work include reviewing, generating, and/or using QALY-based cost-effectiveness evidence; *Role*: Review Industry: I review QALY-based cost-effectiveness evidence submitted by industry or contractors; Review Public: I review publicly available QALY-based cost-effectiveness evidence; Primary study: I conduct primary studies to generate QALY-based cost-effectiveness evidence; Recommend method: I develop or recommend methods for generating QALY-based cost-effectiveness evidence; I do none of the above. One person from the Commonwealth and Central/ Eastern Europe each did not disclose their gender; *Health technology appraised:* Public health professionals refer to appraisals in which workforce capacity or staffing models constituted the object of comparison.

Total number of responses from each region and country: Asia = 95 (China:4, India:5, Indonesia:6, Japan:3, Malaysia:9, Philippines:3, Singapore:15, South Korea:16, Taiwan:11, Thailand:5, Vietnam:18), Central/Eastern Europe = 25 (Bulgaria:6, Croatia:1, Czech Republic:1, Estonia:1, Hungary:5, Latvia:1, Poland:3, Romania:1, Slovenia:6), Western Europe = 31 (Austria:3, Denmark:4, Italy:2, Netherlands:6, Portugal:6, Spain:7, Sweden:3), Latin America = 38 (Argentina:2, Brazil:10, Chile:3, Colombia:12, Ecuador:6, Mexico:3, Peru:2), Middle East/ Africa = 15 (Egypt:2, Saudi Arabia:1, South Africa:5, Tunisia:3, UAE:4), Commonwealth = 34 (Australia:7, Canada:4, England:17, New Zealand:4, Scotland:1, Wales:1).

### Use and importance of utility instruments

Overall, the top three most frequently used utility instruments by HTA practitioners involved with QALY-related work (*n* = 213) were the EQ-5D (“very often”), SF-6D (“occasionally”), and EQ-5D-Y (“occasionally”) (Table 2). Other instruments respondents have come across during their HTA-related work are listed in Supplementary Table 2. Regionally, the use frequency trend was consistent with a few exceptions. In Western Europe, the use frequency for the EQ-5D-Y was “never,” while in Latin America, it was “often” for SF-6D (Supplementary Table 3).

**Table 2.** Median (IQR) responses by region

	Response Frequency, Median (IQR)						Total
	Common-wealth (*n* = 6)	Western Europe (*n* = 7)	Central/Eastern Europe (*n* = 9)	Asia (*n* = 11)	Latin America (*n* = 7)	Middle-East/Africa (*n* = 5)	
Utility Instrument (UI) use frequency							
Total responses (*N*)	33	27	22	83	33	13	211
AQOL	1 (0)	1 (0)	1 (1.0)	1 (1.5)	1 (1.5)	1 (0.5)	6.0
EQ-5D	4 (0)	4 (0)	4 (0)	4 (0)	3.5 (1.0)	4 (0.5)	23.5
EQ-5D-Y	1.75 (0.5)	1 (1)	2 (1.5)	2 (1.0)	2 (1.0)	2 (0.5)	10.75
EQ-HWB	1 (0)	1 (0)	1 (0.75)	1 (0)	1 (1.0)	1 (0.5)	6.0
Bolt-ons	1 (0)	1 (0)	1 (0.25)	1 (0)	1 (0)	1 (0)	6.0
HUI	2 (0.5)	1 (1.0)	1 (1)	1.5 (1.0)	1 (0)	2 (0.5)	8.5
PROPR	1 (0)	1 (0)	1 (0)	1 (0)	1 (0)	1 (0.5)	6.0
QWB	1 (0)	1 (0)	1 (0.5)	1 (0)	1 (0)	1 (0)	6.0
SF-6D	2 (0)	2 (1.0)	2 (1.0)	2 (1.0)	3 (1.0)	2.5 (1.0)	13.5
UI used matters, *n*(%)							
Yes	31 (91.2)	28 (90.3)	22 (88.0)	83 (88.3)	33 (89.2)	10 (66.7)	207 (87.7)
No/ not sure	3 (8.8)	3 (9.7)	3 (12.0)	11 (11.7)	4 (10.8)	5 (33.3)	29 (12.3)
Elicitation Method (EM) use frequency							
Total responses (*N*)	33	27	21	78	32	11	202
BWS	1 (0)	1 (0)	1.5 (2.0)	1 (1.0)	1.5 (1.0)	2 (0.5)	8.0
DCE	2 (0.5)	1.5 (1.0)	2 (1.0)	2 (1.5)	2 (1.0)	3.5 (1.75)	13.0
PTO	1.25 (1.0)	1 (1.0)	2 (0.5)	2 (2.0)	2 (0.5)	2 (1.0)	10.25
SG	2.25 (1.0)	2 (1.0)	2.75 (1.5)	3 (1.0)	3 (1.0)	3.5 (1.5)	16.5
TTO	4 (1.0)	3.5 (1.0)	3 (1.0)	3 (1.0)	3 (1.0)	3.5 (1.0)	20.0
VAS	2 (1.0)	2 (1.0)	3 (1.5)	3 (1.5)	3 (1.5)	3.5 (1.0)	16.5
EM used matters, *n* (%)							
Yes	27 (79.4)	26 (86.7)	16 (69.6)	71 (81.6)	27 (75.0)	11 (84.6)	178 (79.8)
No/ not sure	7 (20.6)	4 (13.3)	7 (30.4)	16 (18.4)	9 (25.0)	2 (15.4)	45 (20.2)
Health Preference Source (HPS) use frequency							
Total responses (*N*)	33	27	22	84	34	13	213
General population own	3.25 (2.0)	3 (2.0)	2 (1.0)	3 (1.5)	2 (0.5)	1 (1.0)	14.25
General population other	2.5 (1.0)	2 (2.0)	4 (1.0)	3 (1.0)	3 (1.0)	3 (1.5)	17.5
Patient own	2 (0)	2 (1.0)	1 (1.0)	3 (1.0)	2 (1.0)	1 (1.0)	11.0
Patient other	2 (2.0)	2 (0.5)	3 (1.5)	2 (1.0)	3 (1.0)	3 (1.5)	15.0
HPS used matter, *n* (%)							
Yes	30 (88.2)	30 (96.8)	23 (92.0)	86 (90.5)	35 (92.1)	13 (86.7)	217 (91.2)
No/ not sure	4 (11.8)	1 (3.2)	2 (8.0)	9 (9.5)	3 (7.9)	2 (13.3)	21(8.9)
Data quality issue frequency							
Total responses (*N*)	34	31	25	95	38	15	238
Patient samples	3 (2.0)	3 (1.5)	2 (0)	3 (1.0)	3 (2.0)	2.5 (1.0)	16.5
Health states	3 (1.0)	2 (1.5)	2 (1.0)	3 (0)	4 (1.0)	3 (1.0)	17.0
Sample size	3 (0.5)	2 (1.0)	3 (1.0)	3 (0)	3 (1.0)	2 (1.0)	16.0
Old data	2 (0)	2 (0.5)	2 (1.25)	2 (1.0)	2 (2.0)	2.5 (0.5)	12.5
Different methods	2.75 (1.0)	2 (1.0)	3 (1.0)	3 (1.0)	3 (2.0)	3 (1.5)	16.75

Abbreviations: *Responses*: 1: Never/not sure; 2: Occasionally; 3: Often; 4: Very often; *Elicitation Methods: BWS:* best-worst scaling; *DCE*: discrete choice experiment; *PTO:* person trade-off; *SG:* standard gamble; *TTO:* time trade-off; *VAS:* visual analogue scale; *Health preference source*: Refers to general population/ patients of one’s own country or other country; *Data quality issue*: *Patient samples*: The patient samples from which HRQoL/utility data was collected were inappropriate (e.g. poor representativeness); *Health states*: The health states (e.g. the vignettes) for which utility data was available do not match the health states in the CEA model; *Sample size*: The population samples from which HRQoL/utility data was collected were too small; *Old data*: The HRQoL/utility data was too old; *Different methods*: The utility values of different health states used in the same model were derived using different methods/instruments.

Countries in each region: Asia (China, India, Indonesia, Japan, Malaysia, Philippines, Singapore, South Korea, Taiwan, Thailand, Vietnam), Central/Eastern Europe (Bulgaria, Croatia, Czech Republic, Estonia, Hungary, Latvia, Poland, Romania, Slovenia), Western Europe (Austria, Denmark, Italy, Netherlands, Portugal, Spain, Sweden), Latin America (Argentina, Brazil, Chile Colombia, Ecuador, Mexico, Peru), Middle East/ Africa (Egypt, Saudi Arabia, South Africa, Tunisia, UAE), Commonwealth (Australia, Canada, England, New Zealand, Scotland, Wales).

Respondents across the regions (Table 3) generally agreed that the choice of utility instrument matters (87.7 percent), ranging from 66.7 percent (Middle East/ Africa) to 91.2 percent (Western Europe). Content analysis revealed that the EQ-5D (EQ-5D-3L/ EQ-5D-5L) instrument was most often selected as the more fit-for-purpose instrument (Table 3) mainly due to its low respondent burden, good psychometric properties, availability of value sets, HTA guide recommendations, and its wide usage that promotes comparability and consistency in the HTA setting.

**Table 3.** More fit-for-purpose tool and their pros and cons and data source issues encountered

Instruments	Counts	Pros	Cons
EQ-5D (EQ-5D-3L/ EQ-5D-5L)	148	Guidelines, value sets, comparability/ consistency in use, widely used, low respondent burden, familiarity, validated/ good psychometric properties, established in HTA	Not sensitive in certain health conditions
Depends	37	Population/condition, standardisation/ consistency/ comparability	
SF-6D	18	Value sets, comparability, established in HTA	
HUI/ HUI3	14	More discriminative in certain conditions, value sets	Expensive, seldom used
AQoL	13	More discriminative in certain conditions	
EQ-5D-Y	13	Child population	
Bolt-ons	7	Improves sensitivity of EQ–5D in certain health conditions	
EQ-HWB	3	Broader quality of life outcomes	Limited experience, insufficient evidence currently (psychometric properties, value sets)
QWB	3	Focus on well-being	Limited experience
CHU-9D	2	Use in child population, value sets.	
PROMIS 10	1	Government use, value sets	
QLQ-C30	1	Value sets, disease specific	

Methods	Counts	Pros	Cons
Time trade-off	55	Valid method, ease of use, involves trade-offs, strong theoretical foundation, produces cardinal utilities	High cognitive burden
Depends	47	Depends on disease area, context, available evidence	
Standard gamble	29	Involves trade-offs, strong theoretical foundation, produces cardinal utilities	High cognitive burden
Discrete choice experiment	28	Ease of understanding, involves trade-offs, online feasibility	Latent utilities, and converting them to quality-adjusted life years-based values
Visual analogue scale	24	Low respondent burden	Not-choice based
Best-worst scaling	4	Ease of understanding	
Person trade-off	4		Low familiarity

Issue	Counts	Description	
Data validity	15	Whose data is more valid (poor quality own country or high-quality other country), logical inconsistencies (the sick have higher values than the general public/ less sick), clinical significance of minute differences observed in disutilities, selection of healthier patients, use of adult values for children	
Generalisability	13	Cross-culture, representativeness of different sub-groups within the country	
Data availability	11	Country-level data, norms, rare disease	
Data collection	9	Timing of interview, short follow-ups, long-term change not considered	
Technique appropriateness	9	Mapping, expert elicitation	
Data analysis	6	Suboptimal methods to obtain utilities, combining utilities of comorbidities, age adjustments, extrapolation to longer time horizon	
Poor reporting	4	Source of utilities, modelling of data, methods to derive utilities	
Data appropriateness	3	Use of other available data	
Utility responsiveness	3	Sensitivity to change and adaptation to disease	
Feasibility	3	Missing values	
Economic evaluation technique concerns	2	Cost-utility analysis is not used to inform decision making, familiarity of decision makers to available techniques	
Validity of quality-adjusted life years in economic evaluations	2	Equity issues	
Study design	2	Real world data, single arm trials	
Content validity	1	Dimensions included in a measure	
Technical	1	Decimal point of utility for cost-utility analysis models	

### Use and importance of preference elicitation methods

The top three most frequently used utility elicitation methods were TTO, VAS, and SG, all of which were “often” used (Table 2). Across regions, the TTO was either “often” or “very often” used to inform decision-making. SG and VAS were only “occasionally” used in Western Europe and the Commonwealth. DCE was “occasionally” used in most regions, but its frequency ranged from “often” and “very often” in the Middle East/Africa. The use frequency of BWS and PTO ranged between “never” and “occasionally” in all regions (Supplementary Table 4).

Respondents across the regions generally agreed that the choice of elicitation method matters (79.8 percent), ranging from 69.6 percent (East and Central Europe) to 86.7 percent (Western Europe). A total of fifty-five respondents mentioned the TTO method as the more fit-for-purpose elicitation method (Table 3). Common reasons (Table 3) included that the TTO had a strong theoretical foundation, involved trade-offs, produced cardinal utilities, was a validated method, and was easy to use. However, the high cognitive burden of the method was often recognized as a limitation of the technique. Many respondents (*n* = 47) mentioned that the elicitation method would need to depend on the disease area, the context of the study, and the availability of evidence.

### Use and importance of health preference data sources

The general public of another country was more frequently used (“often”) than the preferences of the local public (“occasionally”) (Table 2). Region-wise, in Western Europe and the Commonwealth countries, the general population of one’s own country was most frequently used (“often”). In Asia, the general public (own and other countries) and patients (own country) had parallel high usage (“often”). In Latin America, the Middle East/Africa, and Central/Eastern Europe, data of other countries (both general population and patient values) were “often” or “very often” used while the values of one’s own country were only “occasionally” or even “never” utilized (Supplementary Table 5).

Respondents across the regions had a strong consensus that the health preference data source matters (91.2 percent), ranging from 86.7 percent (Middle East/Africa) to 96.8 percent (Western Europe). Of the 144 qualitative responses received, 81 respondents mentioned that the general public’s preferences should determine the utility values. Commonly cited reasons include HTA/country guide’s recommendations, consistency reasons, taxpayers being the most appropriate in a publicly funded healthcare system, and the tendency of patients to adapt to disease, thereby underestimating the disutilities. Conversely, fifty-three respondents felt that patients’ preferences should determine the values, as they reflect the patient voice and capture the disease experience better. Ten people felt it should come from both, either combining both preferences or using them to address different research questions. A total of seventy-nine respondents mentioned that these preferences should come from one’s own country population, as they reflect the culture and context of the preferences more accurately. None explicitly preferred utility values from other countries over their own.

### Use of data with quality concerns

Data quality issues that were “often” encountered across regions included poor sample representativeness, small sample size, poor matching of available data with that needed for CEA models, and data used in the same CEA model generated from multiple elicitation methods/utility instruments (Table 2). These concerns were generally shared across regions. The issue with using outdated data was less of a concern, with most regions reporting it only “occasionally” (Supplementary Table 6).

### Research priorities

Table 4 depicts the research priority by country and region. The top three research priorities globally were i) to make more recent utility values available (recent tariff, importance score (IS) = 0.20), ii) to develop utility instruments to capture the impact of treatment on children and adolescents (children, 0.19), and iii) to develop utility instruments to capture both healthcare and social care impact (social care, 0.17). In Asia (importance score, 0.21) and Western Europe (0.33), the top-voted research priority was related to social care. In the Middle East/Africa (0.33) and Central/Eastern Europe (0.31), the primary research priority was related to recent tariffs. In the Commonwealth (0.23), the priority was to develop utility instruments to capture the impact of treatment on carers; In Latin America (0.22), the top research topic was to develop utility instruments addressing inequality in care. In all regions except for Western Europe and Latin America, children were the second highest research priority (IS = 0.18 to 0.30). In Asia (0.17) and Western Europe (0.15), recent tariffs remain the third highest research priority.

**Table 4.** Research priority by mean sum score

Country	Social Care	Children	Care-givers	Health Specificity	Recent Tariff	Care Inequality	Minority/ rural
Asia (*n* = 11)	**0.21**	**0.18**	**0.09**	**0.15**	**0.17**	**0.13**	**0.08**
China	0.25	0.17	0.00	0.17	0.17	0.17	0.08
India	0.27	0.07	0.00	0.13	0.33	0.20	0.00
Indonesia	0.20	0.13	0.07	0.13	0.20	0.13	0.13
Japan	0.22	0.22	0.11	0.11	0.22	0.00	0.11
Malaysia	0.17	0.17	0.08	0.13	0.13	0.29	0.04
Philippines	0.22	0.22	0.11	0.11	0.00	0.00	0.33
Singapore	0.16	0.20	0.13	0.13	0.31	0.04	0.02
South Korea	0.19	0.27	0.08	0.22	0.22	0.02	0.00
Taiwan	0.28	0.12	0.07	0.17	0.20	0.07	0.10
Thailand	0.13	0.20	0.20	0.20	0.00	0.27	0.00
Vietnam	0.17	0.19	0.12	0.10	0.13	0.20	0.10
Central/Eastern Europe (*n* = 8)	**0.10**	**0.30**	**0.09**	**0.12**	**0.31**	**0.05**	**0.03**
Bulgaria	0.17	0.22	0.06	0.11	0.11	0.06	0.28
Croatia	0.33	0.33	0.00	0.00	0.33	0.00	0.00
Czech Republic	0.00	0.33	0.33	0.00	0.33	0.00	0.00
Hungary	0.00	0.22	0.00	0.28	0.39	0.11	0.00
Latvia	0.00	0.50	0.00	0.00	0.50	0.00	0.00
Poland	0.00	0.42	0.17	0.17	0.25	0.00	0.00
Romania	0.00	0.33	0.00	0.33	0.33	0.00	0.00
Slovenia	0.33	0.00	0.17	0.06	0.22	0.22	0.00
Commonwealth (*n* = 6)	**0.07**	**0.21**	**0.23**	**0.20**	**0.15**	**0.05**	**0.10**
Australia	0.14	0.14	0.10	0.38	0.05	0.05	0.14
Canada	0.08	0.08	0.17	0.17	0.17	0.17	0.17
England	0.17	0.15	0.31	0.13	0.17	0.06	0.02
New Zealand	0.00	0.25	0.17	0.17	0.17	0.00	0.25
Scotland	0.00	0.33	0.33	0.00	0.33	0.00	0.00
Wales	0.00	0.33	0.33	0.33	0.00	0.00	0.00
Western Europe (*n* = 7)	**0.33**	**0.13**	**0.12**	**0.10**	**0.15**	**0.17**	**0.01**
Austria	0.39	0.22	0.00	0.00	0.17	0.22	0.00
Denmark	0.17	0.13	0.13	0.00	0.17	0.42	0.00
Italy	0.33	0.00	0.17	0.17	0.33	0.00	0.00
Netherlands	0.42	0.06	0.17	0.11	0.00	0.25	0.00
Portugal	0.19	0.25	0.11	0.25	0.08	0.06	0.06
Spain	0.28	0.11	0.17	0.06	0.17	0.22	0.00
Sweden	0.50	0.11	0.11	0.11	0.17	0.00	0.00
Latin America (*n* = 7)	**0.16**	**0.08**	**0.15**	**0.16**	**0.11**	**0.22**	**0.12**
Argentina	0.17	0.00	0.17	0.17	0.00	0.33	0.17
Brazil	0.07	0.10	0.15	0.18	0.18	0.22	0.10
Chile	0.22	0.11	0.28	0.11	0.00	0.28	0.00
Colombia	0.17	0.14	0.11	0.25	0.08	0.17	0.08
Ecuador	0.22	0.11	0.22	0.22	0.06	0.11	0.06
Mexico	0.28	0.11	0.11	0.00	0.28	0.11	0.11
Peru	0.00	0.00	0.00	0.17	0.17	0.33	0.33
Middle East/ Africa (*n* = 5)	**0.12**	**0.23**	**0.14**	**0.03**	**0.33**	**0.12**	**0.03**
Egypt	0.17	0.00	0.17	0.17	0.17	0.33	0.00
Saudi Arabia	0.00	0.33	0.33	0.00	0.33	0.00	0.00
South Africa	0.13	0.13	0.07	0.00	0.37	0.17	0.13
Tunisia	0.17	0.42	0.00	0.00	0.42	0.00	0.00
UAE	0.11	0.28	0.11	0.00	0.39	0.11	0.00
Total Average Score	**0.17**	**0.19**	**0.14**	**0.13**	**0.20**	**0.12**	**0.06**

*Note*: Social care: To develop utility instruments to capture the impact of both health care and social care; Children: To develop utility instruments to capture the impact of treatment on children and adolescents; Caregivers: To develop utility instruments that capture the impact of a treatment on carers and caregivers; Health Impact: To develop utility instruments that capture the impact of treatment on more specific aspects of health (e.g. vision hearing etc.); Recent Tariff: To make more recent utility data and value sets/tariffs available; Care Inequality: To develop utility instruments that can address inequality in care; Minority/Rural: To develop utility instruments that can reflect the health preferences of minority groups (e.g. indigenous populations) or rural population.

For example, respondent A from Thailand endorsed two research priorities; social care and children. Each of these two topics received a score of 0.5 and remain topics received a score of 0. To calculate the score for the social care research priority for Thailand, these scores belonging to the individuals from Thailand were averaged. If 10 respondents from Thailand endorsed at least one topic, and the score for social care was 0.5 for five respondents and 0.2 for the remaining five respondents, the importance score for social care in Thailand is 0.35 (i.e., ([0.5 x 5] + [0.2 x 5]) / 10).

## Discussion

In this study, we obtained global insights into the practices, views, and needs of HTA agency personnel across six regions on a broad range of topics related to the measurement and valuation of health. Additionally, we explored data quality issues encountered by HTA practitioners and identified research topics perceived as important.

In general, the respondents’ practices regarding the choice of utility instruments and elicitation methods were consistent with HTA guideline recommendations of using indirect measures such as the EQ-5D instrument as the reference case, and preferring choice-based preference elicitation methods (5;17;18).

Interestingly, only in Western Europe and the Commonwealth were local public health-state preferences used more frequently than those of foreign sources, possibly highlighting the prevalent issue of data availability in the field of HTA (22;23). Additionally, patient preference data is only occasionally used in Western Europe and the Commonwealth, aligning with most HTA guideline recommendations and empirical study findings (24;25). However, patient preference data is often used in regions outside Western Europe and the Commonwealth, perhaps motivated by interest in patients’ views and/or unavailability of preference data from the general public. While respondents’ views regarding the choice of instrument, methods, and health preference data sources generally reflect HTA guidance recommendations, some preferred patient preferences, arguing that the patient voice and disease experience are important. Additionally, some respondents expressed concerns about the shortcomings of the widely used EQ-5D and the TTO method. The main disadvantage of the EQ-5D, as cited by respondents, is poor responsiveness in certain health conditions, while TTO poses a high cognitive burden to respondents. Similar concerns have been documented in the literature (26–28). Interestingly, those respondents did not consider using new instruments such as EQ-HWB, PROPr, “bolt-ons” or new valuation methods such as DCE, perhaps because they were not aware of or familiar with those new alternatives.

This study identified widespread suboptimal use of HRQoL and HSU data in current HTA practice across regions. Data-related issues included sample representativeness, sample size, use of mismatched data, and data generated using different instruments and methods. These issues are likely due to the scarcity of quality and appropriate data, underscoring the need for research to improve data availability (29). Respondents’ comments suggested that HTA practitioners are aware of the data quality issues and the validity of the methods used to address them. This finding echoes the increasing concerns about the methodological rigor in using HSU data for CUA (30–32). However, the magnitude of such issues is largely unknown. A recently conducted systematic review of published CUAs from Asia found that the overall reporting quality for HRQoL or HSU data was very poor (33).

Regarding research priorities, developing instruments to capture the impact of treatments on children and adolescents emerged as an important topic in most regions. This may reflect a real unmet need for fit-for-purpose instruments all around the world. Instruments assessing effects of social care, caregiver needs, and specific health problems are at the top of the wish list of HTA practitioners from many regions. The need for recent tariffs globally as the top research priority further strengthens the importance of valuation work. This need was especially emphasized in the Middle East/Africa and Central/Eastern Europe, where value set generation is only starting to gain momentum (34–36). Countries in these regions generally lack preference-based values. In line with the growth of HTA in these countries, the presence of value sets becomes essential in expanding the use of CUAs and in implementing HTA for wider coverage of healthcare decision-making (37–39). An interesting research topic proposed by a respondent is to develop public depositories of HSU data. Such a depository would act as a library, storing data from different population groups, facilitating crosswalks to other country value sets, and being referenced by HTA practitioners as needed. A properly regulated HSU depository would alleviate the issue of scarce data faced by HTA practitioners globally. These research topics, along with insights from the content analysis, highlight the global need for greater generation of HSUs in different areas to better capture the health preferences of populations.

The above findings on the current practice and views of HTA personnel on instruments, methods, and data for generating QALY-based evidence may provide useful guidance for future research. First, research on instruments targeting children and adolescents, such as EQ-5D-Y, may be prioritized due to a global need for such tools. Compared to HRQoL instruments for adults, those for children and adolescents are fewer and less developed (40). The methods review completed by NICE in 2022 found insufficient evidence for recommending any existing HRQoL instruments for use in pediatric HTA and therefore called for research in this area (41). Second, researchers developing new instruments and elicitation methods may consider shifting from a pure academic approach to a user-oriented approach by engaging stakeholders, particularly HTA agencies, in the whole development process. Such a collaborative approach may increase the chance of developing a product that will be accepted or adopted sooner for use in HTA practice. This approach to involving stakeholders such as patient advocacy groups and decision-makers has been used in developing the EQ-HWB instrument (42). Given that instrument development is a lengthy, multi-stage process and HTA agencies are cautious in endorsing new instruments and methods, sustained engagement and long-term collaboration may be necessary to achieve a tangible impact. Moreover, given that established HTA agencies are more concerned with maintaining consistency and standardization, new instruments and methods may be more likely to be endorsed and accepted by burgeoning HTA agencies. Last but not least, research on methods for making more HRQoL and HSU data available or making better use of existing data seems equally or even more important than making new instruments available (see Table 5, which summarizes respondent-identified priority research topics related to utility values, many of which concern data availability). This is because data scarcity for endorsed HRQoL instruments such as EQ-5D may be a greater issue than the lack of more fit-for-purpose instruments, such as EQ-HWB, because those instruments are routinely used. Such research may involve systematically collecting and publishing HRQoL data from health systems, collating and compiling HRQoL data published in the literature, and developing tools for modifying or transforming HRQoL data for use across health systems. Databases providing HSU data and guidelines promoting appropriate use of HSU data (32;43) have been available. However, those may not be sufficient and more work is needed to fill in this data gap and need.

**Table 5.** Other research topic of importance-related to utility values

Research topic
To assess the validity of quality-adjusted life years in capturing outcomes
To capture productivity losses and double counting with quality of life measures
To develop a public depository of health-state utilities
To develop guidelines on instrument use to increase comparability
To develop patient-specific utilities
To ensure instruments used have content validity- relevant domains/health states are captured
To generate living health-state utilities over long time horizons
To generate utility values of rare disease
To make country-specific utility data available
To make cross-country preference and utility evidence available
To produce more research on the impact of shifting to EQ-5D-5L instrument

This study had several limitations. In twelve of the forty-five countries, there were fewer than three responses despite repeated reminders, limiting the representativeness of these countries. A further limitation is the difficulty in identifying the most appropriate respondent within an HTA agency. Senior personnel may be well-positioned to speak on behalf of the organisation, but may not possess detailed knowledge of the specific research area. Conversely, analysts may have relevant expertise but may not be able to represent the agency’s official position, for example, due to limited seniority or differing personal views. Another limitation concerns the snowball-type recruitment method we employed. Potential respondents were identified through the network of EuroQol Group members. As a result, HTA personnel who are familiar with or favor EuroQol instruments may be overrepresented in our sample. However, EuroQol Group members come from diverse backgrounds and regions, and many actively participate in HTA development in their respective countries, making them ideal recruiters for this by-invitation-only global survey. A related limitation relates to our recruitment strategy. Because recruitment was organized at the country level rather than at the level of specific HTA bodies, respondents were encouraged to provide their views as individuals rather than as formal representatives of their organizations. Consequently, we were unable to identify respondents by specific HTA agency within each country and therefore could not reanalyze the data using the HTA body as the unit of analysis. Although we considered additional subgroup analyses, for example, examining differences by type of technology appraised or therapeutic area, we judged that such analyses would not yield reliable or interpretable findings. Many respondents reported involvement across multiple therapeutic areas, multiple health technology types, or held several HTA roles, making clear classification challenging. Moreover, the study was not powered to detect meaningful subgroup differences, and uneven distribution across categories would further limit the validity of any conclusions drawn. Lastly, we were not able to verify the eligibility of the respondents. Country-specific survey links were distributed by recruiters to potential respondents in the target HTA agencies. Although screening questions were included at the start of the survey, personal identifiers such as respondent names or the agencies they worked for were not collected to encourage more candid responses.

## Conclusions

This study addresses important knowledge gaps regarding the current practices of measuring and valuing HRQoL in HTA and the views on the challenges and needs of HTA agency personnel worldwide. Findings from this study may guide research aimed at developing tools and methods for providing high-quality QALY evidence for economic evaluations.

## Supporting information

10.1017/S0266462326103602.sm001 Vasan Thakumar et al. supplementary material Vasan Thakumar et al. supplementary material

## Data Availability

Data generated for the current study are included in this published article (and its supplementary files). They are also available from the corresponding author on reasonable request.

## Supplementary material

The supplementary material for this article can be found at http://doi.org/10.1017/S0266462326103602.
