# Supplementary material for: What do HTA agencies need for generating health-related quality of life evidence? Findings from a global survey
Source: Int J Technol Assess Health Care. 2026 Feb 27;42(1):e33. doi: 10.1017/S0266462326103602 (PMC13071849; doi:10.1017/S0266462326103602)
Supplement: Vasan Thakumar et al. supplementary material [file S0266462326103602sup001.docx]

**Title:** What do HTA agencies need for generating health-related quality of life evidence? Findings from a global survey

**Table of contents**

[Supplementary Information on Methods: 1](#_Toc219493689)

[Supplementary Table 1 Responses by country 3](#_Toc219493690)

[Supplementary Table 2 Other instruments respondents have come across during their HTA related work 4](#_Toc219493691)

[Supplementary Table 3 Utility instrument use frequency 4](#_Toc219493692)

[Supplementary Table 4 Elicitation method use frequency 6](#_Toc219493693)

[Supplementary Table 5 Frequency of using data from different sources 8](#_Toc219493694)

[Supplementary Table 6 Data quality issue frequency: Patient samples, health states, sample size, old data, different methods 10](#_Toc219493695)

# Supplementary Information on Methods:

Specifications of the survey form

Section One inquired about the frequency of using or reviewing data collected from nine MAUIs, including AQOL, EQ-5D (EQ-5D-3L/ EQ-5D-5L), EQ-5D-Y, EQ-HWB, “bolt-ons” (not an instrument, per say, but questions used to adapt MAUIs), HUI, PROPr, QWB, and SF-6D. Section Two addressed the frequency of using or reviewing data collected using six elicitation methods including best-worst scaling (BWS), DCE, person trade-off (PTO), standard gamble (SG), TTO, and visual analogue scale (VAS). In Section Three, we surveyed the frequency of using preference data from different sources, including data from the general population of one’s own country or other countries, and data from patients of one’s own country or other countries.

Section Four covered the frequency of encountering five concerns related to HRQoL/HSU data, while in Section Five, respondents rated the importance of seven specific research topics related to HSUs, selecting up to three (of the seven research topics) they felt important. The final section assessed the participants’ demographic and professional characteristics.

Statistical analysis of nominated research priorities in Section Five

To analyze the nominated research priorities in Section Five, we excluded respondents (N=16) who did not endorse the importance of any research topics. For each of the remaining respondents who endorsed one to three research topics, each endorsed topic received a score of 1/N (N is the number of topics a respondent endorsed) and each unendorsed topic received a score of 0.

# Supplementary Table 1 Responses by country

|  | Country | Responses |
| --- | --- | --- |
| 1 | **Argentina** | 2 |
| 2 | **Australia** | 7 |
| 3 | **Austria** | 3 |
| 4 | **Belgium** | 0 |
| 5 | **Brazil** | 10 |
| 6 | **Bulgaria** | 6 |
| 7 | **Canada** | 4 |
| 8 | **Chile** | 3 |
| 9 | **China** | 4 |
| 10 | **Colombia** | 12 |
| 11 | **Croatia** | 1 |
| 12 | **Czech Republic** | 1 |
| 13 | **Denmark** | 4 |
| 14 | **Ecuador** | 6 |
| 15 | **Egypt** | 2 |
| 16 | **England** | 17 |
| 17 | **Estonia** | 1 |
| 18 | **Hungary** | 5 |
| 19 | **India** | 5 |
| 20 | **Indonesia** | 6 |
| 21 | **Ireland** | 0 |
| 22 | **Italy** | 2 |
| 23 | **Japan** | 3 |
| 24 | **Latvia** | 1 |
| 25 | **Lithuania** | 0 |
| 26 | **Malaysia** | 9 |
| 27 | **Mexico** | 3 |
| 28 | **Netherlands** | 6 |
| 29 | **New Zealand** | 4 |
| 30 | **Peru** | 2 |
| 31 | **Philippines** | 3 |
| 32 | **Poland** | 3 |
| 33 | **Portugal** | 6 |
| 34 | **Romania** | 1 |
| 35 | **Saudi Arabia** | 1 |
| 36 | **Scotland** | 1 |
| 37 | **Singapore** | 15 |
| 38 | **Slovakia** | 0 |
| 39 | **Slovenia** | 6 |
| 40 | **South Africa** | 5 |
| 41 | **South Korea** | 16 |
| 42 | **Spain** | 7 |
| 43 | **Sweden** | 3 |
| 44 | **Taiwan** | 11 |
| 45 | **Thailand** | 5 |
| 46 | **Tunisia** | 3 |
| 47 | **UAE** | 4 |
| 48 | **Vietnam** | 18 |
| 49 | **Wales** | 1 |
|  | **Country** | **Reason for non-response** |
| 1 | Cuba | Survey was not carried out because of current political turmoil in the country |
| 2 | Finland | Survey was not carried out because potential contact person was non-responsive |
| 3 | France | Survey was not carried out because the HTA agency declined to participate |
| 4 | Germany | Survey was not carried out because Germany does not use CEA to inform country-level decision making |
| 5 | Greece | Survey was not carried out because potential contact person was non-responsive |
| 6 | Hong Kong | Survey was not carried out because Hong Kong does not use CEA for HTA decision making |
| 7 | Israel | Survey was not carried out because of the current political turmoil in the country |
| 8 | Jamaica | Survey was not carried out because HTA is in its infancy in the Caribbean |
| 9 | Norway | Survey was not carried out because of the red tape involved with distributing the survey |
| 10 | Trinidad and Tobago | Survey was not carried out because HTA is in its infancy in the Caribbean |
| 11 | USA | Survey was not carried out because CEA is not frequently used to inform HTA decision making |

# Supplementary Table 2 Other instruments respondents have come across during their HTA related work

| Instrument | Count Overall | Country |
| --- | --- | --- |
| Disease-specific instruments | 11 | Singapore (1), Vietnam (1), Hungary (1), Denmark (1), Netherlands (2), Brazil (2), Colombia (1), Australia (1), England (1) |
| Direct utility elicitation (SG, TTO, VAS, DCE) | 7 | Japan (1), Thailand (1), Thailand (1), Poland (1),Netherlands (1),Australia (1) |
| EORTC QLQ-C30 | 6 | Taiwan (1), Denmark (1), Portugal (1), Colombia (1), Australia (1), England (1) |
| Mapping | 6 | Japan (1), Netherlands (1), Portugal (1), Brazil (1), Australia (1), England (1) |
| CHU9D | 5 | Australia (3); England (1); Wales (1) |
| Health-Related Quality of Life Instrument with 8 Items (HINT-8) | 4 | South Korea |
| Asia PBM 7 | 2 | Thailand |
| Vignettes | 2 | Portugal (1), England (1) |
| PedsQL | 1 | Australia |
| Patient Health Questionnaire-9 (PHQ-9) | 1 | Vietnam |
| Beck Depression Inventory (BDI) | 1 | Australia |
| EORTC QLU-C10D | 1 | Australia |
| Visual Function Questionnaire - Utility Index (VFQ-UI) | 1 | Brazil |
| Asthma Quality of Life Questionnaire (AQLQ) | 1 | Colombia |
| Cambridge Pulmonary Hypertension Outcome Review (CAMPHOR) | 1 | Singapore |
| The Functional Assessment of Cancer Therapy (FACT) | 1 | Colombia |
| Functional Assessment of Cancer Therapy - Lung (FACT-L) | 1 | Australia |
| MOS Social Support Survey | 1 | Vietnam |
| St. George's Respiratory Questionnaire (SGRQ) | 1 | Australia |
| Western Ontario and McMaster Universities Arthritis Index (WOMAC) | 1 | Australia |
| Functional Assessment of Chronic Illness Therapy (FACIT) | 1 | Poland |
| Mini-Mental Adjustment to Cancer (MAC) | 1 | Vietnam |
| Depression Anxiety and Stress Scale 21- Short Form (DASS-21) | 1 | Vietnam |
| Six-minute walking test (6MWT) | 1 | Australia |
| 15D | 1 | Denmark |
| Veterans RAND 12 Item Health Survey (VR-12) | 1 | Canada |
| WHOQOL-BREF | 1 | India |
| ICECAP A and O | 1 | Netherlands |
| Acceptance and Action Questionnaire version 2 (AAQ-2) | 1 | Vietnam |

# Supplementary Table 3 Utility instrument use frequency

| Country | Total responses (N) | Value | Statistic | Value | Statistic | Value | Statistic | Value | Statistic | Value | Statistic | Value | Statistic | Value | Statistic | Value | Statistic | Value | Statistic |
| --- | --- | --- | --- | --- | --- | --- | --- | --- | --- | --- | --- | --- | --- | --- | --- | --- | --- | --- | --- |
|  |  | AQOL | | EQ-5D | | EQ-5D-Y | | EQ-HWB | | Bolt-ons | | HUI | | PROPR | | QWB | | SF6D | |
| Argentina | 2 | 3 | mode | 3 | mode | 3 | mode | 3 | mode | 1 | mode | 2 | p50 | 1 | mode | 1 | mode | 3 | p50 |
| Australia | 6 | 2 | mode | 4 | mode | 1.5 | p50 | 1 | mode | 1 | mode | 3 | p50 | 1 | mode | 1 | mode | 2.5 | p50 |
| Austria | 1 | 1 | mode | 4 | mode | 1 | mode | 1 | mode | 1 | mode | 1 | mode |  | nil | 1 | mode | 2 | mode |
| Brazil | 10 | 1 | mode | 4 | mode | 2 | mode | 1 | mode | 1 | mode | 1 | mode | 1 | mode | 1 | mode | 3 | p50 |
| Bulgaria | 6 | 1 | mode | 4 | mode | 2 | mode | 2 | mode | 1 | mode | 1 | mode | 1 | mode | 1.5 | p50 | 4 | mode |
| Canada | 4 | 1 | mode | 4 | mode | 1 | mode | 1 | mode | 1 | mode | 2.5 | p50 | 1 | mode | 1 | mode | 2 | mode |
| Chile | 3 | 2 | mode | 4 | mode | 2 | mode | 1 | mode | 1 | mode | 1 | mode | 1 | mode | 1 | mode | 3 | mode |
| China | 4 | 2.5 | p50 | 4 | mode | 1 | mode | 1 | mode | 2 | mode | 1 | mode | 1 | mode | 1 | mode | 3 | mode |
| Colombia | 10 | 1 | mode | 4 | mode | 1 | mode | 1 | mode | 1 | mode | 1 | mode | 1 | mode | 1 | mode | 3 | mode |
| Croatia | 1 | 1 | mode | 4 | mode |  | nil | 1 | mode | 1 | mode | 1 | mode | 1 | mode | 1 | mode | 2 | mode |
| Czech Republic | 1 |  | nil | 4 | mode |  | nil | 1 | mode | 1 | mode | 2 | mode |  | nil |  | nil | 2 | mode |
| Denmark | 4 | 1 | mode | 4 | mode | 1.5 | p50 | 1 | mode | 1 | mode | 1 | mode | 1 | mode | 1.5 | p50 | 2 | mode |
| Ecuador | 4 | 1 | mode | 3 | p50 | 2 | mode | 2 | mode | 1 | mode | 1 | mode | 1 | mode | 2 | p50 | 2 | mode |
| Egypt | 2 | 1 | mode | 4 | mode | 3 | mode | 2 | mode | 2 | mode | 2 | mode | 2 | mode | 1 | mode | 2.5 | p50 |
| England | 17 | 1 | mode | 4 | mode | 2 | mode | 1 | mode | 2 | mode | 2 | mode | 1 | mode | 1 | mode | 2 | mode |
| Estonia | 1 |  | nil | 4 | mode |  | nil |  | nil |  | nil | 2 | mode |  | nil |  | nil | 3 | mode |
| Hungary | 5 | 2 | mode | 4 | mode | 2.5 | p50 | 1 | mode | 1 | mode | 2 | mode | 1 | mode | 1 | mode | 2 | mode |
| India | 5 | 3 | mode | 4 | mode | 4 | mode | 3 | mode | 2 | p50 | 3 | mode | 2 | mode | 3 | mode | 3 | p50 |
| Indonesia | 6 | 1 | mode | 4 | mode | 1 | mode | 1 | mode | 1 | mode | 1 | mode | 1 | mode | 1 | mode | 2 | p50 |
| Italy | 1 | 1 | mode | 4 | mode | 2 | mode | 1 | mode | 1 | mode | 1 | mode | 1 | mode | 1 | mode | 2 | mode |
| Japan | 3 | 1 | mode | 4 | mode | 2 | mode | 1 | mode | 1 | mode | 2 | mode | 1 | mode | 1 | mode | 2 | p50 |
| Latvia | 1 | 1 | mode | 4 | mode | 1 | mode | 1 | mode | 1 | mode | 2 | mode | 1 | mode | 1 | mode | 2 | mode |
| Malaysia | 8 | 1.5 | p50 | 4 | mode | 2 | mode | 1 | mode | 1 | mode | 2 | mode | 1 | mode | 1 | mode | 3 | mode |
| Mexico | 2 | 2.5 | p50 | 3.5 | p50 | 2 | p50 | 1 | mode | 1 | mode | 1 | mode | 1 | mode | 1 | mode | 2.5 | p50 |
| Netherlands | 6 | 1 | mode | 4 | mode | 1 | mode | 1 | mode | 1 | mode | 2 | p50 | 1.5 | p50 | 1 | mode | 3 | mode |
| New Zealand | 4 | 1 | mode | 4 | mode | 1.5 | p50 | 1 | mode | 1 | mode | 2 | mode | 1 | mode | 1 | mode | 2 | mode |
| Peru | 2 | 1 | mode | 3.5 | p50 | 3 | mode | 1 | mode | 1 | mode | 1 | mode | 1 | mode | 1 | mode | 2 | p50 |
| Philippines | 3 | 1 | mode | 4 | mode | 2 | p50 | 1 | mode | 1 | mode | 1.5 | p50 | 1 | mode | 1 | mode | 2 | mode |
| Poland | 3 | 1.5 | p50 | 4 | mode | 3 | mode | 2 | p50 | 1.5 | p50 | 1 | p50 | 2 | mode | 2 | mode | 4 | mode |
| Portugal | 6 | 1 | mode | 4 | mode | 1 | mode | 1 | mode | 1 | mode | 1 | mode | 1 | mode | 1 | mode | 2 | mode |
| Romania | 1 | 1 | mode | 1 | mode | 1 | mode | 1 | mode | 1 | mode | 1 | mode | 1 | mode | 1 | mode | 1 | mode |
| Saudi Arabia | 1 | 1 | mode | 4 | mode | 2 | mode | 1 | mode | 1 | mode | 2 | mode | 1 | mode | 2 | mode | 3 | mode |
| Scotland | 1 | 1 | mode | 4 | mode | 2 | mode | 1 | mode | 1 | mode | 2 | mode | 1 | mode | 1 | mode | 2 | mode |
| Singapore | 14 | 1 | mode | 4 | mode | 1 | mode | 1 | mode | 1 | mode | 1 | mode | 1 | mode | 1 | mode | 2 | p50 |
| Slovenia | 3 | 2.5 | p50 | 3 | mode | 2 | mode | 1.5 | p50 | 2 | mode | 1 | mode | 1 | mode | 1 | mode | 2.5 | p50 |
| South Africa | 3 | 1 | mode | 3.5 | p50 | 1 | mode | 1 | mode | 1 | mode | 1.5 | p50 | 1 | mode | 1 | mode | 2 | mode |
| South Korea | 16 | 1 | mode | 4 | mode | 2 | p50 | 1 | mode | 1 | mode | 1 | mode | 1 | mode | 1 | mode | 2 | mode |
| Spain | 6 | 1 | mode | 4 | mode | 2.5 | p50 | 1 | mode | 1 | mode | 1 | mode | 1 | mode | 1 | mode | 3 | mode |
| Sweden | 3 | 1 | mode | 4 | mode | 1 | mode | 1 | mode | 1 | mode | 2 | mode | 1 | mode | 1 | mode | 2 | mode |
| Taiwan | 11 | 1 | mode | 4 | mode | 2 | p50 | 1 | mode | 1 | mode | 1 | mode | 1 | mode | 1 | mode | 3 | p50 |
| Thailand | 5 | 1 | mode | 4 | mode | 2 | mode | 1 | mode | 1 | mode | 2 | mode | 1 | mode | 1 | mode | 2 | mode |
| Tunisia | 3 |  | nil | 4 | mode | 2 | p50 | 1.5 | p50 | 1 | mode | 2.5 | p50 | 1 | mode | 1 | mode | 3 | p50 |
| UAE | 4 | 2 | p50 | 2 | p50 | 1.5 | p50 | 1 | mode | 1 | mode | 1 | mode | 1.5 | p50 | 1 | mode | 1 | mode |
| Vietnam | 8 | 3 | mode | 3.5 | p50 | 3 | p50 | 1 | mode | 1 | mode | 1.5 | p50 | 1 | mode | 1 | mode | 3 | mode |
| Wales | 1 | 1 | mode | 4 | mode | 4 | mode | 1 | mode | 1 | mode | 2 | mode | 1 | mode | 1 | mode | 2 | mode |
| MEDIAN (IQR) |  | 1 | (1-1.5) | 4 | (4-4) | 2 | (1-2) | 1 | (1-1) | 1 | (1-1) | 1.5 | (1-2) | 1 | (1-1) | 1 | (1-1) | 2 | (2-3) |

Notes: p50: median; The number of responses column reflects IQR for median responses. Responses: 1: Never; 2: Occasionally; 3: Often; 4:Very often

# Supplementary Table 4 Elicitation method use frequency

| Country | N | Value | Statistic | Value | Statistic | Value | Statistic | Value | Statistic | Value | Statistic | Value | Statistic |
| --- | --- | --- | --- | --- | --- | --- | --- | --- | --- | --- | --- | --- | --- |
|  |  | BWS | | DCE | | PTO | | SG | | TTO | | VAS | |
| Argentina | 2 | 1.5 | p50 | 1.5 | p50 | 2.5 | p50 | 2.5 | p50 | 2.5 | p50 | 2.5 | p50 |
| Australia | 6 | 1.5 | p50 | 4 | mode | 1.5 | p50 | 2 | mode | 3 | mode | 2 | mode |
| Austria | 1 |  | nil |  | nil | 2 | mode | 2 | mode | 4 | mode | 2 | mode |
| Brazil | 10 | 2 | mode | 3 | mode | 1 | mode | 3 | mode | 4 | mode | 4 | mode |
| Bulgaria | 6 | 1 | mode | 1 | mode | 2 | p50 | 2 | mode | 2.5 | p50 | 4 | mode |
| Canada | 4 | 1 | mode | 1 | mode | 1 | mode | 2.5 | p50 | 4 | mode | 1 | mode |
| Chile | 3 | 2 | mode | 3 | mode | 2.5 | p50 | 3 | p50 | 3 | p50 | 4 | mode |
| China | 4 | 2 | mode | 3.5 | p50 | 2 | mode | 2 | mode | 4 | mode | 2.5 | p50 |
| Colombia | 10 | 2 | mode | 2 | mode | 2 | mode | 3 | mode | 3 | p50 | 4 | mode |
| Croatia | 1 |  | nil | 3 | mode |  | nil |  | nil |  | nil |  | nil |
| Czech Republic | 1 |  | nil | 2 | mode |  | nil | 2 | mode | 2 | mode | 2 | mode |
| Denmark | 4 | 1 | mode | 1 | mode | 1 | mode | 1 | mode | 3 | p50 | 2.5 | p50 |
| Ecuador | 4 | 1 | mode | 2 | mode | 2 | p50 | 3 | mode | 3 | p50 | 2 | mode |
| Egypt | 2 | 3.5 | p50 | 3 | p50 |  | nil | 3 | p50 | 3 | p50 | 4 | mode |
| England | 17 | 1 | mode | 2 | mode | 1 | mode | 3 | mode | 4 | mode | 1 | mode |
| Estonia | 1 | 3 | mode | 2 | mode | 3 | mode | 4 | mode | 4 | mode | 4 | mode |
| Hungary | 5 | 2 | mode | 2 | mode | 1.5 | p50 | 3 | mode | 3 | p50 | 3 | p50 |
| India | 5 | 2 | mode | 4 | mode | 4 | mode | 4 | mode | 4 | mode | 4 | mode |
| Indonesia | 6 | 1 | mode | 1 | mode | 2 | p50 | 2 | mode | 2 | p50 | 4 | mode |
| Italy | 1 | 1 | mode | 1 | mode | 1 | mode | 2 | mode | 2 | mode | 3 | mode |
| Japan | 3 | 1 | mode | 4 | mode | 1 | mode | 2 | mode | 3 | p50 | 1 | mode |
| Latvia | 1 | 1 | mode |  | nil |  | nil | 3 | mode | 3 | mode | 3 | mode |
| Malaysia | 8 | 3 | mode | 2 | mode | 2 | p50 | 4 | mode | 3 | mode | 4 | mode |
| Mexico | 2 | 1 | mode | 2 | p50 | 2 | mode | 2 | mode | 2 | mode | 3 | mode |
| Netherlands | 6 | 1 | mode | 2 | mode | 1 | mode | 2 | mode | 3.5 | p50 | 2 | mode |
| New Zealand | 4 | 1 | mode | 2.5 | p50 | 2 | mode | 2 | mode | 2.5 | p50 | 4 | mode |
| Peru | 2 | 1 | mode | 2.5 | p50 | 2 | mode | 1.5 | p50 | 2 | p50 | 2.5 | p50 |
| Philippines | 3 | 1.5 | p50 | 3 | mode | 3 | mode | 3 | mode | 2 | mode | 3 | mode |
| Poland | 3 | 3 | p50 | 2 | mode | 2 | p50 | 4 | mode | 3 | p50 | 4 | mode |
| Portugal | 6 | 1 | mode | 1.5 | p50 | 1.5 | p50 | 3 | p50 | 4 | mode | 3 | p50 |
| Romania | 1 | 1 | mode | 1 | mode | 1 | mode | 1 | mode | 1 | mode | 2 | mode |
| Saudi Arabia | 1 | 2 | mode | 4 | mode | 2 | mode | 4 | mode | 4 | mode | 4 | mode |
| Scotland | 1 | 1 | mode | 2 | mode | 1 | mode | 2 | mode | 4 | mode | 2 | mode |
| Singapore | 14 | 1 | mode | 2 | mode | 1 | mode | 3 | mode | 3 | mode | 3 | p50 |
| Slovenia | 3 | 1.5 | p50 | 1 | mode | 2 | p50 | 2.5 | p50 | 3.5 | p50 | 3 | mode |
| South Africa | 3 | 1.5 | p50 | 1.5 | p50 | 1 | mode | 2 | mode | 3 | p50 | 3 | p50 |
| South Korea | 16 | 1 | mode | 3 | mode | 1 | mode | 3 | mode | 3 | mode | 3 | mode |
| Spain | 6 | 1 | mode | 4 | mode | 2 | p50 | 4 | mode | 4 | mode | 2 | p50 |
| Sweden | 3 | 1 | mode | 1.5 | p50 | 1 | mode | 3 | mode | 3 | p50 | 2 | mode |
| Taiwan | 11 | 1 | mode | 1 | mode | 3 | mode | 2 | mode | 2 | mode | 4 | mode |
| Thailand | 5 | 1 | mode | 2 | mode | 1 | mode | 2 | mode | 2 | mode | 2 | mode |
| Tunisia | 3 | 1 | mode | 4 | mode | 2 | mode | 4 | mode | 4 | mode | 3.5 | p50 |
| UAE | 4 | 2 | mode |  | nil |  | nil |  | nil |  | nil | 2 | mode |
| Vietnam | 8 | 1 | mode | 2 | mode | 2 | p50 | 3 | p50 | 2.5 | p50 | 4 | mode |
| Wales | 1 | 1 | mode | 2 | mode | 2 | mode | 3 | mode | 4 | mode | 2 | mode |
| MEDIAN (IQR) |  | 1 | (1-2) | 2 | (1.5-3) | 2 | (1-2) | 3 | (2-3) | 3 | (2.5-4) | 3 | (2-4) |

Notes: p50: median; The number of responses column reflects IQR for median responses. Responses: 1: Never; 2: Occasionally; 3: Often; 4:Very often, BWS: best-worst scaling; DCE: discrete choice experiment; PTO: person trade-off; SG: standard gamble; TTO: time trade-off; VAS: visual analogue scale;

# Supplementary Table 5 Frequency of using data from different sources

| Country | N | Value | Statistic | Value | Statistic | Value | Statistic | Value | Statistic |
| --- | --- | --- | --- | --- | --- | --- | --- | --- | --- |
|  |  | General Pop Own Country | | General Pop Other Country | | Patient Own Country | | Patient Other Country | |
| Argentina | 2 | 2 | mode | 3 | mode | 2 | mode | 3 | mode |
| Australia | 6 | 2.5 | p50 | 2 | mode | 3 | mode | 3 | mode |
| Austria | 1 | 1 | mode | 4 | mode | 2 | mode | 2 | mode |
| Brazil | 10 | 2 | p50 | 4 | mode | 2 | mode | 4 | mode |
| Bulgaria | 6 | 2 | mode | 4 | mode | 2 | mode | 4 | mode |
| Canada | 4 | 4 | mode | 3 | mode | 2 | mode | 2 | mode |
| Chile | 3 | 3 | mode | 4 | mode | 2 | mode | 3 | mode |
| China | 4 | 3 | mode | 3 | p50 | 3 | mode | 2 | mode |
| Colombia | 10 | 2 | p50 | 3 | p50 | 2 | mode | 3 | mode |
| Croatia | 1 | 1 | mode | 3 | mode | 1 | mode | 3 | mode |
| Czech Republic | 1 | 1 | mode | 4 | mode | 1 | mode | 4 | mode |
| Denmark | 4 | 4 | mode | 2 | mode | 2 | mode | 1.5 | p50 |
| Ecuador | 5 | 2 | p50 | 4 | mode | 1 | mode | 4 | mode |
| Egypt | 2 | 1 | mode | 2.5 | p50 | 1 | mode | 4 | mode |
| England | 17 | 4 | mode | 2 | mode | 2 | mode | 1 | mode |
| Estonia | 1 | 1 | mode | 4 | mode | 1 | mode | 2 | mode |
| Hungary | 5 | 2 | mode | 4 | mode | 1 | mode | 3.5 | p50 |
| India | 5 | 3 | p50 | 2 | mode | 4 | mode | 2 | mode |
| Indonesia | 6 | 3.5 | p50 | 3 | mode | 3 | p50 | 3 | mode |
| Italy | 1 | 2 | mode | 2 | mode | 2 | mode | 2 | mode |
| Japan | 3 | 4 | mode | 3 | p50 | 2 | p50 | 2 | p50 |
| Latvia | 1 | 1 | mode | 4 | mode | 1 | mode | 3 | mode |
| Malaysia | 8 | 2 | mode | 3 | p50 | 2 | mode | 3 | p50 |
| Mexico | 2 | 2.5 | p50 | 2 | mode | 1.5 | p50 | 2 | p50 |
| Netherlands | 6 | 3 | mode | 2 | mode | 1 | mode | 2 | mode |
| New Zealand | 4 | 2 | mode | 3 | mode | 2 | mode | 3 | mode |
| Peru | 2 | 1 | mode | 3 | p50 | 1 | mode | 3.5 | p50 |
| Philippines | 3 | 2 | mode | 3 | mode | 2 | mode | 3 | mode |
| Poland | 3 | 3 | mode | 3 | mode | 2.5 | p50 | 3.5 | p50 |
| Portugal | 6 | 4 | mode | 2 | mode | 1 | mode | 2 | p50 |
| Romania | 1 | 2 | mode | 2 | mode | 2 | mode | 2 | mode |
| Saudi Arabia | 1 | 2 | mode | 4 | mode | 2 | mode | 3 | mode |
| Scotland | 1 | 4 | mode | 2 | mode | 2 | mode | 1 | mode |
| Singapore | 15 | 2 | p50 | 4 | mode | 1 | mode | 2 | mode |
| Slovenia | 3 | 3 | mode | 2 | mode | 2 | mode | 2 | p50 |
| South Africa | 3 | 1 | mode | 3 | p50 | 1 | mode | 2.5 | p50 |
| South Korea | 16 | 4 | mode | 2 | mode | 3 | mode | 2 | mode |
| Spain | 6 | 4 | mode | 4 | mode | 4 | mode | 3 | p50 |
| Sweden | 3 | 2 | p50 | 3 | p50 | 2 | mode | 2.5 | p50 |
| Taiwan | 11 | 2 | mode | 3 | mode | 2 | mode | 3 | mode |
| Thailand | 5 | 3 | p50 | 2 | p50 | 4 | mode | 2 | p50 |
| Tunisia | 3 | 1 | mode | 4 | mode | 1 | mode | 4 | mode |
| UAE | 4 | 2 | mode | 2 | mode | 2 | p50 | 1.5 | p50 |
| Vietnam | 8 | 3 | mode | 2 | mode | 3 | mode | 2 | mode |
| Wales | 1 | 2 | mode | 4 | mode | 2 | mode | 2 | mode |
| MEDIAN (IQR) |  | 2 | (2-3) | 3 | (2-4) | 2 | (2-3) | 1 | (1-1) |

Notes: p50: median; The number of responses column reflects IQR for median responses. Responses: 1: Never; 2: Occasionally; 3: Often; 4: Very often; Health preference source: Refers to general population/ patients of one’s own country or other country

# Supplementary Table 6 Data quality issue frequency: Patient samples, health states, sample size, old data, different methods

| Country | N | Value | Statistic | Value | Statistic | Value | Statistic | Value | Statistic | Value | Statistic |
| --- | --- | --- | --- | --- | --- | --- | --- | --- | --- | --- | --- |
|  |  | Patient samples | | Health states | | Sample size | | Old data | | Different methods | |
| Argentina | 2 | 2 | mode | 2 | mode | 3 | mode | 1 | mode | 2 | mode |
| Australia | 7 | 2 | mode | 3 | mode | 2 | mode | 2 | mode | 3 | p50 |
| Austria | 3 | 2 | mode | 2 | mode | 2 | mode | 1.5 | p50 | 2 | mode |
| Brazil | 10 | 4 | mode | 4 | mode | 3 | mode | 2.5 | p50 | 4 | mode |
| Bulgaria | 6 | 2 | mode | 3 | p50 | 2 | mode | 3 | p50 | 2 | mode |
| Canada | 4 | 4 | mode | 3 | mode | 3 | mode | 2.5 | p50 | 2 | mode |
| Chile | 3 | 4 | mode | 4 | mode | 3 | p50 | 3 | mode | 3 | mode |
| China | 4 | 3 | mode | 3 | mode | 3 | mode | 3 | mode | 3 | mode |
| Colombia | 12 | 4 | mode | 3 | mode | 3 | mode | 3 | mode | 3 | mode |
| Croatia | 1 | 3 | mode | 3 | mode | 3 | mode | 3 | mode | 3 | mode |
| Czech Republic | 1 | 2 | mode | 2 | mode | 3 | mode |  | nil | 2 | mode |
| Denmark | 4 | 4 | mode | 2 | mode | 2 | mode | 2 | mode | 1 | mode |
| Ecuador | 6 | 3 | p50 | 4 | mode | 2 | mode | 2 | p50 | 2 | p50 |
| Egypt | 2 | 2.5 | p50 | 3 | p50 | 1.5 | p50 | 2.5 | p50 | 3 | mode |
| England | 17 | 3 | mode | 2 | mode | 3 | mode | 2 | mode | 2 | mode |
| Estonia | 1 | 2 | mode | 3 | mode | 2 | mode | 2 | mode | 3 | mode |
| Hungary | 5 | 2 | mode | 2 | mode | 3 | mode | 2 | mode | 3 | p50 |
| India | 5 | 3 | mode | 3 | mode | 3 | mode | 3 | mode | 3 | mode |
| Indonesia | 6 | 3 | mode | 2.5 | p50 | 3 | mode | 2 | mode | 2 | p50 |
| Italy | 2 | 3 | p50 | 1.5 | p50 | 1.5 | p50 | 2 | p50 | 3 | mode |
| Japan | 3 | 3 | mode | 4 | mode | 3 | mode | 2 | mode | 3 | mode |
| Latvia | 1 | 2 | mode | 2 | mode | 1 | mode | 1 | mode | 3 | mode |
| Malaysia | 9 | 2 | p50 | 3 | mode | 3 | p50 | 3 | mode | 2 | mode |
| Mexico | 3 | 3 | mode | 3 | mode | 1 | mode | 1 | mode | 3 | mode |
| Netherlands | 6 | 3 | mode | 3 | mode | 3 | mode | 2 | p50 | 4 | mode |
| New Zealand | 4 | 2 | mode | 2 | mode | 2.5 | p50 | 2 | mode | 2.5 | p50 |
| Peru | 2 | 2 | mode | 4 | mode | 2 | mode | 1.5 | p50 | 4 | mode |
| Philippines | 3 | 4 | mode | 3 | mode | 3 | mode | 2 | mode | 3 | mode |
| Poland | 3 | 3 | mode | 2 | mode | 3 | p50 | 2 | p50 | 3 | mode |
| Portugal | 6 | 2.5 | p50 | 2.5 | p50 | 2.5 | p50 | 1.5 | p50 | 2 | mode |
| Romania | 1 | 2 | mode | 2 | mode | 2 | mode | 1 | mode |  | nil |
| Saudi Arabia | 1 | 4 | mode | 4 | mode | 4 | mode | 4 | mode | 4 | mode |
| Scotland | 1 | 3 | mode | 3 | mode | 3 | mode | 2 | mode | 3 | mode |
| Singapore | 15 | 2 | mode | 3 | mode | 3 | mode | 3 | mode | 3 | mode |
| Slovenia | 6 | 2 | mode | 2 | mode | 3 | mode | 2.5 | p50 | 2 | mode |
| South Africa | 5 | 3 | p50 | 3 | mode | 2 | mode | 2 | p50 | 2 | mode |
| South Korea | 16 | 2 | mode | 3 | mode | 2 | mode | 2 | mode | 3 | mode |
| Spain | 7 | 4 | mode | 3.5 | p50 | 2 | mode | 3 | mode | 3 | mode |
| Sweden | 3 | 3 | mode | 1 | mode | 3 | mode | 1 | mode | 2 | mode |
| Taiwan | 11 | 3 | p50 | 3 | p50 | 3 | p50 | 2 | mode | 2 | mode |
| Thailand | 5 | 2 | mode | 2 | mode | 3 | mode | 2 | mode | 3 | mode |
| Tunisia | 3 | 2 | mode | 2 | p50 | 3 | mode | 2.5 | p50 | 3.5 | p50 |
| UAE | 4 | 2 | mode | 2 | mode | 2 | mode | 2 | mode | 2 | mode |
| Vietnam | 18 | 2 | mode | 3 | mode | 3 | p50 | 2 | mode | 3 | p50 |
| Wales | 1 | 4 | mode | 4 | mode | 4 | mode | 2 | mode | 4 | mode |
| MEDIAN (IQR) | | 3 | (2-3) | 3 | (2-3) | 3 | (2-3) | 2 | (2-2.5) | 3 | (2-3) |

**Abbreviations:** p50: median; The number of responses column reflects IQR for median responses. Responses: 1: Never; 2: Occasionally; 3: Often; 4:Very often; Patient samples: The patient samples from which HRQoL/utility data was collected were inappropriate (e.g. poor representativeness); Health states: The health states (e.g. the vignettes) for which utility data was available do not match the health states in the CEA model; Sample size: The population samples from which HRQoL/utility data was collected were too small; Old data: The HRQoL/utility data was too old; Different methods: The utility values of different health states used in the same model were derived using different methods/instruments
